# Supplementary material for: Will Absolute Risk Estimation for Time to Next Screen Work for an Asian Mammography Screening Population?
Source: Cancers (Basel). 2023 Apr 29;15(9):2559. doi: 10.3390/cancers15092559 (PMC10177032; doi:10.3390/cancers15092559)
Supplement: Supplementary file 1 [file cancers-15-02559-s001.zip › cancers-2357624-supplementary.pdf]

Supplementary Table S1. Model performance indicators of the Gail model when applied to 26,380 women. AUC: Area under the ROC Curve, CI: confidence interval, RR: the relative risk estimates associated with breast cancer risk factors from the BCRA package ("Whites" or "Asian-American" population).

(RR/Incidence and mortality rates)	x-year absolute risk	Observed (O)	Expected (E)	E/O (95% CI)	AUC (95% CI)
White/White	2	37	124.28	3.36 (2.43 - 4.64)	0.600 (0.513 - 0.687)
	5	150	325.09	2.17 (1.85 - 2.54)	0.586 (0.539 - 0.633)
	10	403	687.33	1.71 (1.55 - 1.88)	0.584 (0.556 - 0.613)
	15	633	1060.01	1.67 (1.55 - 1.81)	0.580 (0.557 - 0.603)
	Lifetime at aged 80 years	1000	1621.24	1.62 (1.52 - 1.72)	0.595 (0.577 - 0.612)
Asian-American/ Asian-American	2	37	73.16	1.98 (1.43 - 2.73)	0.605 (0.514 - 0.696)
	5	150	186.46	1.24 (1.06 - 1.46)	0.601 (0.552 - 0.650)
	10	403	368.47	0.91 (0.83 - 1.01)	0.602 (0.574 - 0.631)
	15	633	543.59	0.86 (0.79 - 0.93)	0.589 (0.566 - 0.612)
	Lifetime at aged 80 years	1000	815.43	0.82 (0.77 - 0.87)	0.592 (0.574 - 0.610)
Asian-American/ Asian-American	2	37	95.06	2.57 (1.86 - 3.55)	0.628 (0.536 - 0.720)
	5	150	242.83	1.62 (1.38 - 1.90)	0.595 (0.546 - 0.645)
	10	403	496.57	1.23 (1.12 - 1.36)	0.596 (0.567 - 0.625)
	15	633	741.52	1.17 (1.08 - 1.27)	0.590 (0.567 - 0.614)
	Lifetime at aged 80 years	1000	1114.21	1.11 (1.05 - 1.19)	0.596 (0.578 - 0.614)

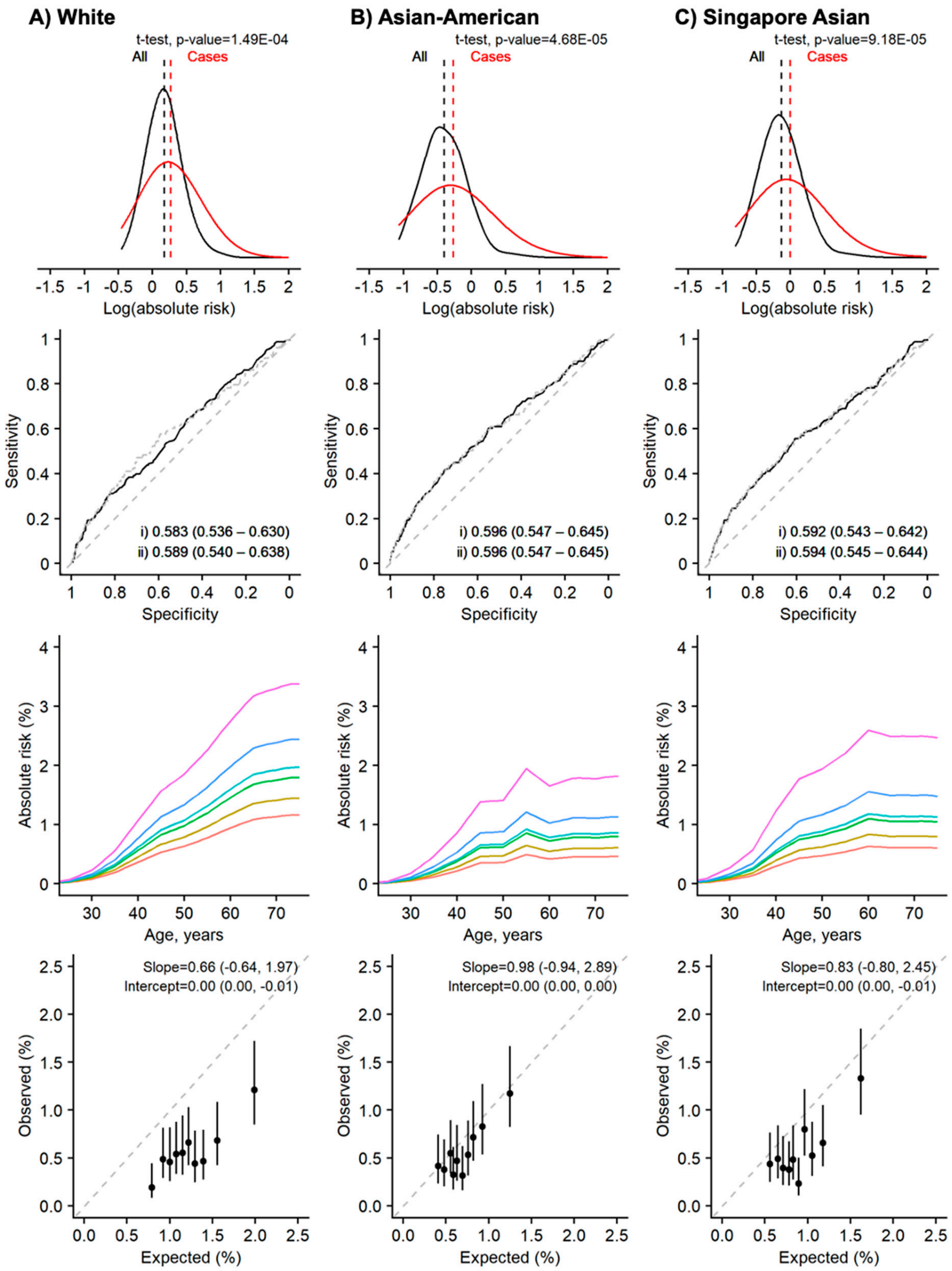

Supplementary Figure S1. The Gail model (5-year absolute risk) performance assessment. *Using the relative risk estimates from the White population [column A], the closest Asian populations [columns B and C]. The BCRA package's breast cancer incidence rates and mortality rates were replaced with the rates from Singapore's population for the right-most panel [column C]. Rows (from top) 1) distribution, 2) discrimination, 3) predictive ability, and 4) calibration by deciles. Two-sided, two-sample t-tests with a type I error of 0.05 were used to examine whether there was a difference in the distribution of the log absolute risk between breast cancer cases and the entire population (row 1). The Area Under the Receiver Operator Characteristic Curve (AUC) values, i) unadjusted (solid line), and ii) adjusted for age at recruitment (dashed line) (row 2). The coloured lines (from bottom to top) in the plots for predictive ability denote 1-, 20-, 60-, 80-, 95-, and 99-percentile of the absolute risk (row 3). Calibration is calculated based on 5-year absolute risk by deciles in (row 4).*

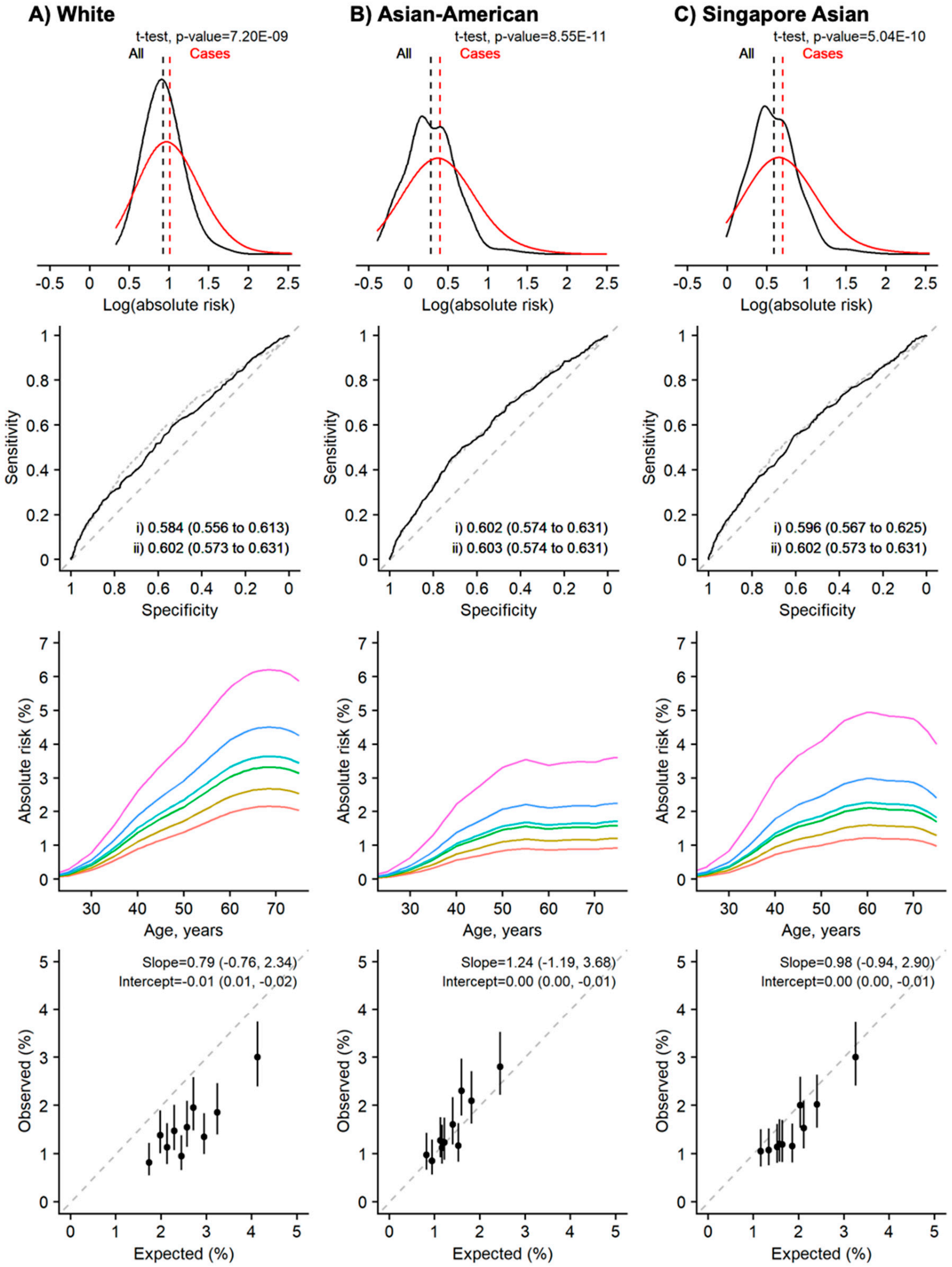

Supplementary Figure S2. The Gail model (10-year absolute risk) performance assessment. *Using the relative risk estimates from the White population [column A], the closest Asian populations [columns B and C]. The BCRA package's breast cancer incidence rates and mortality rates were replaced with the rates from Singapore's population for the right-most panel [column C]. Rows (from top) 1) distribution, 2) discrimination, 3) predictive ability, and 4) calibration by deciles. Two-sided, two-sample t-tests with a type I error of 0.05 were used to examine whether there was a difference in the distribution of the log absolute risk between breast cancer cases and the entire population (row 1). The Area Under the Receiver Operator Characteristic Curve (AUC) values, i) unadjusted (solid line), and ii) adjusted for age at recruitment (dashed line) (row 2). The coloured lines (from bottom to top) in the plots for predictive ability denote 1-, 20-, 60-, 80-, 95-, and 99-percentile of the absolute risk (row 3). Calibration is calculated based on 10-year absolute risk by deciles in (row 4).*

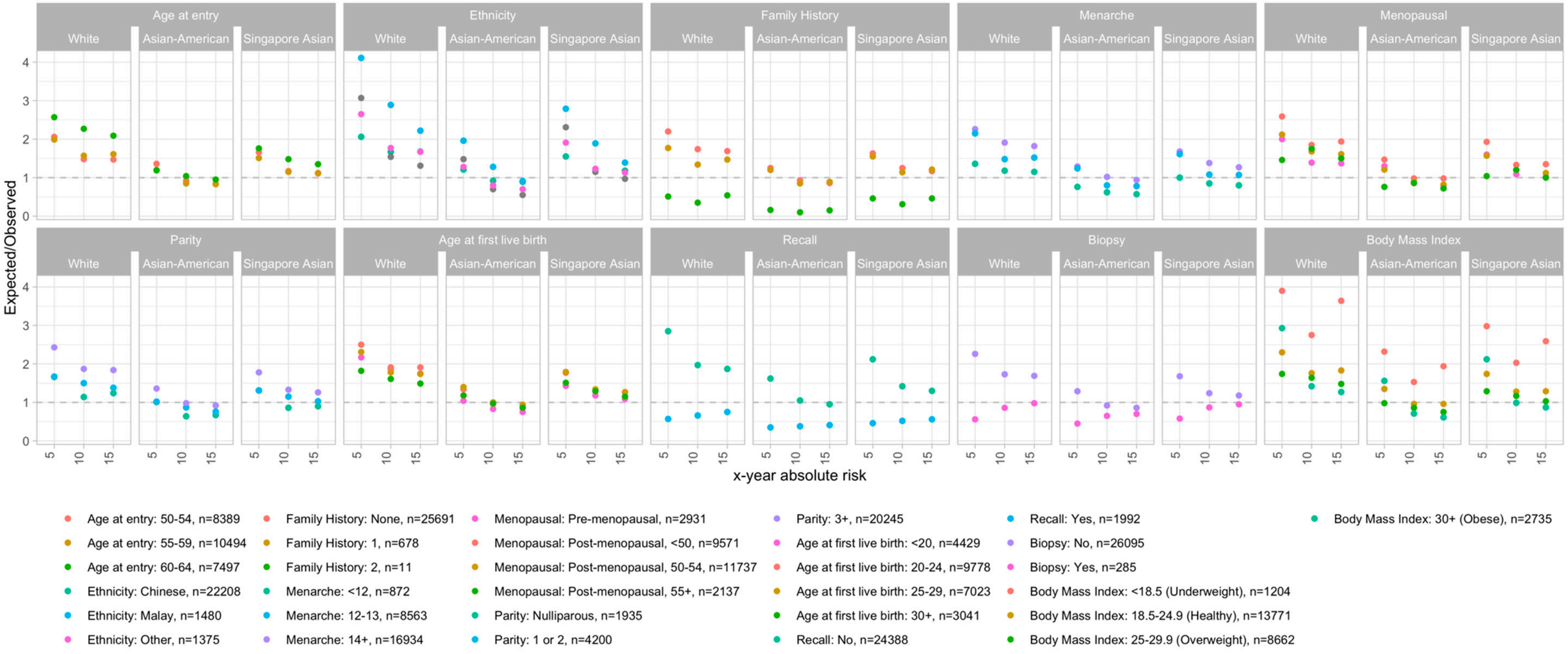

Supplementary Figure S3. Calibration of the Gail model for 5-, 10- and 15-year absolute risks, by subgroups. *Using the relative risk estimates from the White population [White], and the closest Asian populations [Asian-American and Singapore Asian]. The BCRA package's breast cancer incidence rates and mortality rates were replaced with the rates from Singapore's population [Singapore Asian].*
